# Takayasu arteritis presenting as a stroke in young: a case report

**DOI:** 10.1097/MS9.0000000000001582

**Published:** 2024-01-03

**Authors:** Paras Oli, Prabhat Poudel, Shradha KC, Neeraj Thapa, Aastha KC

**Affiliations:** a Department of Internal Medicine (Neurology), Nepal Medical College; b Department of Internal Medicine, Kathmandu Medical College, Kathmandu; c Department of Internal Medicine, Chitwan Medical College, Chitwan, Nepal

**Keywords:** arteritis, stroke, takayasu

## Abstract

**Introduction:**

Takayasu arteritis is a large-vessel vasculitis predominantly seen in young women. Lack of signs and symptoms in the early stage of the disease often delays the diagnosis and thus leads to significant morbidity and mortality. One severe complication that may arise is a significant narrowing of blood vessels, potentially leading to life-threatening ischemic repercussions.

**Case presentation:**

The authors present a case of a 29-year-old female who presented to our ER with features of left-sided hemiparesis and right-sided facial deviation. Computed tomography angiography and Carotid Doppler helped in making the diagnosis of Takayasu arteritis. She was managed with prednisolone and mycophenolate mofetil. She has been on a regular follow-up for the last year and is currently stable.

**Conclusion:**

Even being a rare scenario, stroke can be the initial presentation of Takayasu arteritis. Early diagnosis and management in young patients are vital in keeping the disease at bay and preventing physical, mental, and socio-economic adversities.

## Introduction

Highlights

- Takayasu arteritis is a large-vessel vasculitis commonly seen in young Asian women.
- Stroke can present as an initial presentation in an asymptomatic person with Takayasu arteritis.
- Prompt diagnosis and management are necessary to prevent life-threatening ischemic complications in the future.

Takayasu arteritis is a rare, chronic large-vessel vasculitis commonly involving central large vessels like the aorta and its branches. The early stage of the disease is usually the asymptomatic ‘pre-pulseless’ stage with nonspecific findings that later progress to the ‘pulseless’ stage, manifesting as an ischemic complication, missing pulses, and a history of claudication^1^. It mainly affects young women clinically, presenting with symptoms of headache, joint pain, weight loss, hypertension, angina, heart failure, renal failure, vision loss, postprandial abdominal pain, dizziness, vertigo, amaurosis fugax, and cerebrovascular accidents like transient ischemic attacks (TIAs) and stroke. Almost 10–20% of patients with Takayasu arteritis can present with TIA or stroke^2^. Stroke as an initial presentation of Takayasu arteritis is rare^3^. Here, we report a case of a 29-year-old lady with neglected chronic back pain who presented to us with symptoms of right-sided middle cerebral artery (MCA) stroke as an initial presentation of Takayasu arteritis.

## Case presentation

A 29-year-old female presented to our emergency room with 6 h of left-sided weakness and right-sided facial deviation. Her history was significant for 2 years of chronic back pain in the interscapular region, occasional myalgia, and tingling sensations over the back. She had no history of fever, blurred vision, dizziness, headache, nausea, or vomiting. She has been on amlodipine 5 mg for hypertension for the last 2 years but has no history of diabetes. She does not consume alcohol or cigarettes. There was no family history of cerebrovascular accidents. On examination, she was pale, conscious, oriented, and following commands. Pulse and blood pressure were undetectable in the upper limbs with normal lower limb measurements. Neurological examination revealed left-sided hemiplegia more prominent on the upper limb with the power of ⅖ and ⅗ on the lower limb, hyperreflexia, and an extensor plantar reflex. Weakness of the facial muscles on the left side of the Upper Motor Neuron type was seen. A soft-pitched, early diastolic, decrescendo murmur was heard loudest at the left lower sternal border. A bruit was heard over the bilateral carotid arteries.

**Figure 1:**
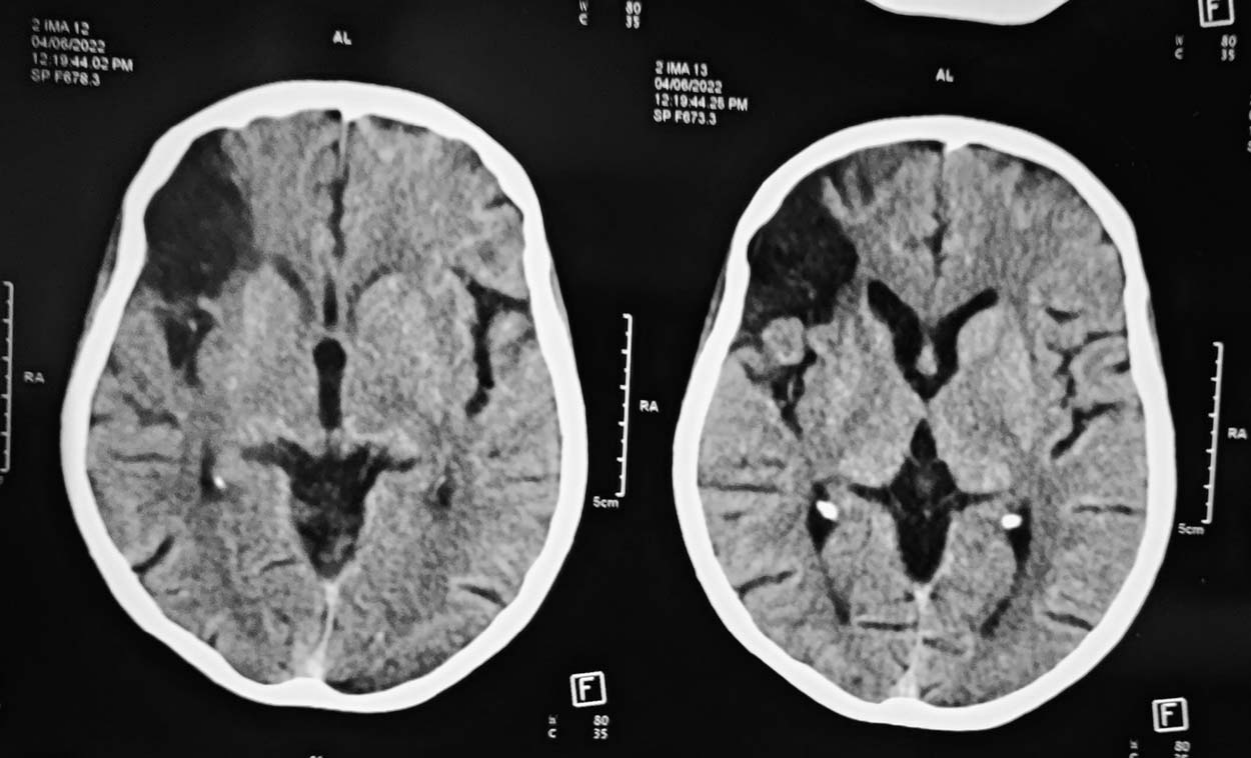
NC-CT scan of head showing encephalomalacic changes in the right anterior frontal lobe region.

Laboratory investigations showed an erythrocyte sedimentation rate of 50 mm/h. Creatinine was 2.9 mg/dl, and urea was 59 mg/dl, which reached baseline after 1 week. The ECG was normal. An emergency noncontrast head computed tomography (CT) scan revealed encephalomalacic changes in the right anterior frontal lobe region (Fig. 1). On further inquiry, the patient’s family reported that the patient had a history of left-sided weakness, slurring of speech, and left facial weakness 1 month ago, for which she did not seek any medical help. The patient was managed conservatively due to late presentation beyond the window period for thrombolysis, and we started with the administration of aspirin 150 mg and atorvastatin 40 mg. Regarding the diagnosis of stroke in young patients, further investigations were carried out to identify the cause. Magnetic resonance angiography (MRA) and T1/T2 weighted MRI of the brain and neck revealed encephalomalacic changes and gliosis in the right anterior frontal lobe with normal internal carotid artery (ICA), MCA, and anterior cerebral artery. Unfortunately, due to financial constraints, further investigations were delayed. CT angiography of the head and neck two months after initial presentation showed subacute right frontal lobe infarct with variable circumferential wall thickening of the arch of the aorta, right brachiocephalic trunk, bilateral subclavian artery, and bilateral common carotid, external carotid, and ICA. Changes were predominantly in the bilateral subclavian artery, proximal left common carotid artery (CCA), and right ICA till the proximal C2 segment, causing their complete nonopacification.

**Figure 2:**
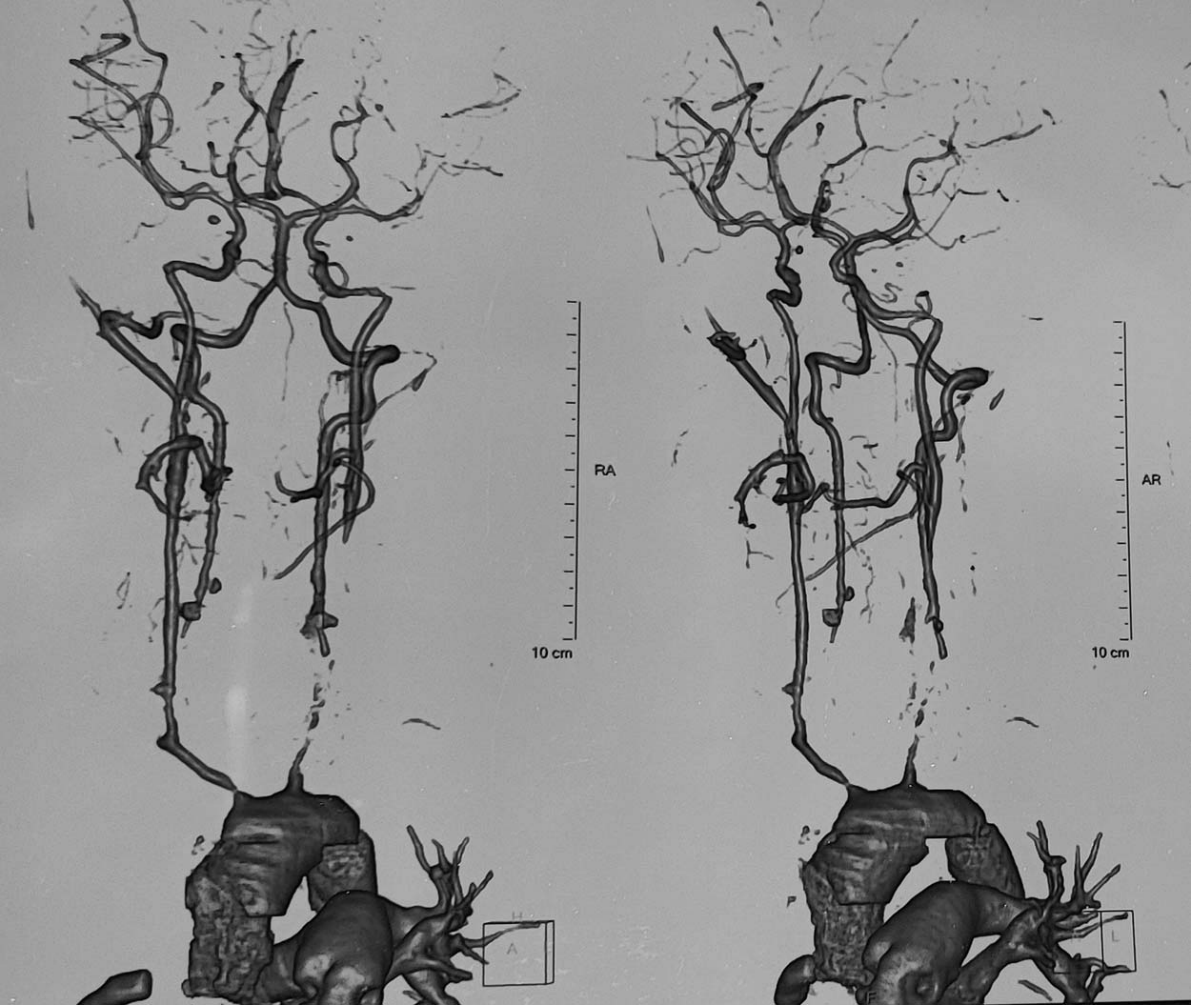
CTA showing severe focal narrowing of right ICA, marked wall thickening of the whole length of the left CCA with severe stenosis.

Transthoracic echocardiography showed a bicuspid aortic valve, mild concentric left ventricular hypertrophy, moderate-severe AR (aortic regurgitation), and grade 1 left ventricular diastolic dysfunction with a preserved ejection fraction of 60%. Takayasu arteritis was diagnosed as per the Modified Ishikawa Criteria for Diagnosis of Takayasu Arteritis^1^. The patient was prescribed aspirin, atorvastatin, metoprolol, amlodipine, tapering dose prednisolone, and mycophenolate mofetil (MMF). The patient was invited to follow-up with us after 2 weeks, and multispecialty care was planned involving a team of rheumatology, neurology, and cardiology. Carotid Doppler done at 2-month follow-up revealed diffusely thickened walls of the bilateral CCA and brachiocephalic trunk with features suggestive of large-vessel vasculitis, severely narrowed lumen of the left CCA with resultant monophasic, very low resistance flow in the left ICA with near total loss of pulsatility in the left ICA, and very low resistance monophasic flow with loss of pulsatility in bilateral vertebral artery suggestive of high-grade proximal stenosis. Follow-up CT angiography of the head and neck in maximum intensity projection after 1 month revealed severe focal narrowing of the right ICA with more than 90% stenosis, marked wall thickening of the whole length of the left CCA with severe stenosis in the proximal portion, and complete occlusion of the mid-CCA along with reconstitution of the right distal CCA from collaterals. Occlusion of bilateral subclavian arteries along with normal bilateral vertebral arteries and left vertebral arteries supplying collaterals in the left subclavian region with the possibility of left subclavian steal syndrome was revealed (Fig. 2). Disease activity was not controlled with conventional disease-modifying antirheumatic drugs( DMARDs) (MMF) at the initial follow-up after 2 weeks. Due to financial constraints, neither biologic DMARDs nor surgery for carotid occlusion were started. Initial treatment was not able to control disease activity, and the patient also started feeling anxious and overwhelmed due to her paralysed status, but with familial support, regular physiotherapy, and monthly follow-up, she is currently stable on prednisolone and MMF.

## Discussion

Takayasu arteritis is a rare chronic granulomatous large-vessel pan-vasculitis commonly affecting young women. It was first described by a Japanese ophthalmologist, Dr Mikito Takayasu, in 1905 as a case presenting with absent pulses and retinal arteriovenous anastomoses^4^. A recent systematic review showed a prevalence of 3.2–40.0 cases per million and an incidence of 0.4–2.6 cases per million per year^1^. Its clinical presentation has a wide range of symptoms, commonly asymptomatic in the early stages, progressing to ischemic complications. Ischemic symptoms can vary according to the vessel’s involvement. Chronic ischemia due to occlusion or stenosis of the aortic branches leads to neurological complications like headache, dizziness, blurred vision, syncope, TIAs, and even stroke, as in our patient. Slow vascular fibrosis allows time for collateral formation to compensate for ischemia. Such patients are diagnosed incidentally on examination with findings of an absent pulse, asymmetrical blood pressure on the limbs, and vascular bruit. Inadequate collateral leads to signs of claudication, whereas abrupt vascular occlusion leads to ischemic complications and end organ damage like stroke and myocardial infarction without any preceding constitutional symptoms^5^.

Stroke commonly occurs due to embolism of the vessel, typically involving the anterior circulation, or can also occur due to thrombotic occlusion of the vessel^6^. Embolus can be of cardiac origin or from distant thrombosed arteries like the carotid. Takayasu arteritis can present with hypertensive emergencies leading to hemorrhagic stroke as well, and stroke as an initial presentation of Takayasu arteritis is common in older patients^5^. Our patient presented with signs and symptoms of an acute MCA stroke, making it an unusual case. The diagnosis of Takayasu arteritis is challenging due to the early asymptomatic phase^1^. So proper physical examination and laboratory investigations play a vital role. Imaging studies play a crucial role in the accurate diagnosis of Takayasu^1^. In our patient, the clinical presentation of stroke and CT findings of ischemic changes with abnormal upper limb pulse and blood pressure, along with computed tomography angiography of the neck and carotids, MRA of the brain and neck, and carotid Doppler, led us toward the diagnosis of Takayasu. MRA has a sensitivity of 90% and a specificity of 80% in the diagnosis of aortic lesions^6^. Clinical assessment and angiographic findings are used as diagnostic criteria. Both the Ishikawa diagnostic criteria of 1988 and the American College of Rheumatology classification criteria of 1990 included an age of less than 40 as a diagnosis criterion, whereas modified Ishikawa criteria not including age increased sensitivity and specificity to 92.5 and 95%, respectively^1^. After ruling out other autoimmune diseases with negative autoimmune antibodies (ANA, dsDNA, and lupus anticoagulant), our case was diagnosed using the Modified Ishikawa Criteria.

Early diagnosis and the start of immunosuppressants with lifelong vascular surveillance and invasive therapy in case of complications have been pillars for the management of Takayasu arteritis^1^. The latest EULAR guidelines for management focus on the early starting of glucocorticoids along with conventional synthetic DMARDs like methotrexate and mycophenolate (Phase I). Response to initial therapy is assessed by EULAR disease activity. Phase I-resistant Takayasu are managed with biologic DMARDs like tocilizumab and TNF inhibitors (Phase II)^1^. A study by showed statin and antiplatelet therapy prevent ischemic complications in Takayasu. Our patient was initially managed as per Phase I guidelines with antiplatelet and statin. The tapering dose of the steroid did not show any improvement, so the dose of MMF was increased to 1 g taken twice a day. The patient did not have any significant adverse effects from the medication. Stenotic lesions of vessels mainly supplying the brain are managed with endarterectomy or angioplasty, and neither was done in our case due to financial limitations.

## Conclusion

Takayasu arteritis can be a debilitating condition if not managed properly. Ischemic complications like stroke and myocardial infarction can be life-threatening. Stroke at a young age carries a lot of challenges in management and being secondary to Takayasu arteritis, and as a first manifestation of the disease, it can be a life-changing incident if not tackled properly. Like our patient, Takayasu arteritis complications can negatively impact a person’s physical, mental, and socio-economic condition as they depend on others for a long duration. So early diagnosis and management of the case are of great significance. The financial constraints of the patient and her family also significantly hampered her care. This calls for changes in policies for the provision of financial support to such patients in low-middle income countries like ours.

## Ethical approval

We are submitting case report for which ethical approval is not required.

## Consent

Written informed consent was obtained from the patient for publication and any accompanying images. A copy of the written consent is available for review by the Editor-in-Chief of this journal on request.

## Sources of funding

None.

## Author contribution

P.O.: literature review, manuscript preparation, and review; P.P., S.K.C., N.T., and A.K.C.: manuscript preparation and review.

## Conflicts of interest disclosure

None.

## Guarantor

Paras Oli.
